# Transcriptional Regulation of the β-Type Carbonic Anhydrase Gene *bca* by RamA in *Corynebacterium glutamicum*

**DOI:** 10.1371/journal.pone.0154382

**Published:** 2016-04-27

**Authors:** Adnan Shah, Bernhard J. Eikmanns

**Affiliations:** Institute of Microbiology and Biotechnology, University of Ulm, D-89069 Ulm, Germany; University of Houston, UNITED STATES

## Abstract

Carbonic anhydrase catalyzes the reversible hydration of carbon dioxide to bicarbonate and maintains the balance of CO_2_/HCO_3_^-^ in the intracellular environment, specifically for carboxylation/decarboxylation reactions. In *Corynebacterium glutamicum*, two putative genes, namely the *bca* (cg2954) and *gca* (cg0155) genes, coding for β-type and γ-type carbonic anhydrase, respectively, have been identified. We here analyze the transcriptional organization of these genes. The transcriptional start site (TSS) of the *bca* gene was shown to be the first nucleotide “A” of its putative translational start codon (ATG) and thus, *bca* codes for a leaderless transcript. The TSS of the *gca* gene was identified as an “A” residue located at position -20 relative to the first nucleotide of the annotated translational start codon of the cg0154 gene, which is located immediately upstream of *gca*. Comparative expression analysis revealed carbon source-dependent regulation of the *bca* gene, with 1.5- to 2-fold lower promoter activity in cells grown on acetate as compared to glucose as sole carbon source. Based on higher expression of *bca* in a mutant deficient of the regulator of acetate metabolism RamA as compared to the wild-type of *C*. *glutamicum* and based on the binding of His-tagged RamA protein to the *bca* promoter region, we here present evidence that RamA negatively regulates expression of *bca* in *C*. *glutamicum*. Functional characterization of a *gca* deletion mutant of *C*. *glutamicum* revealed the same growth characteristics of *C*. *glutamicum* ∆*gca* as that of wild-type *C*. *glutamicum* and no effect on expression of the *bca* gene.

## Introduction

Carbonic anhydrase (CA) (EC 4.2.1.1) catalyzes the reversible hydration of carbon dioxide (CO_2_) to bicarbonate (HCO_3_^-^) and plays an important role in various biochemical and physiological processes in prokaryotic and eukaryotic organisms [1, 2]. CAs are ubiquitously found in eukarya, bacteria and archaea domains of life [2, 3, 4] and five genetically distinct CA families are known to date, namely the α-, β-, γ-, δ-, and ζ-CAs [5]. The α-class is predominant in mammals whereas the δ- and ζ-classes have been found in marine diatoms [6]. The β and γ are the ancient classes of CAs, predominantly found in prokaryotes and their presence in species of archaea and bacteria indicate their fundamental role in prokaryotic biology [6, 7, 8]. The bacterial β-CAs are zinc metalloenzymes that maintain CO_2_/HCO_3_^-^ balance in the intracellular environment [9, 10]. By keeping a given balance, the CAs also represent important “accessory enzymes” for other enzymes that use CO_2_ or HCO_3_^-^ [10], such as ribulose-1,5-bisphosphate carboxylase/oxygenase (RuBisCO) in chloroplasts, carbamate hydro-lyase (cyanase) in *Escherichia coli* [11], urease in *Helicobacter pylori* [12] and HCO_3_^-^-dependent carboxylases in a variety of eukaryotes and prokaryotes [10, 13–17]. In several bacteria, CA has been shown to be essential during aerobic growth under normal atmospheric conditions [14–17] and Ueda et al. [18] suggested that microorganisms that are lacking CA can persist in nature only by choosing niches with higher CO_2_ concentrations.

*Corynebacterium glutamicum* is a Gram-positive, facultative anaerobic organism, able to use a variety of sugars, alcohols, and organic acids as carbon and energy source [19–21]. The organism has a long tradition in biotechnology and is used as an “industrial workhorse” for the production of amino acids, mainly L-glutamate and L-lysine [22, 23]. In addition, the use of *C*. *glutamicum* in the production of other amino acids [24–30], different organic acids [31–34], vitamins [35], diamines [36–40], ethanol and higher alcohols [41–45], 2-ketoacids [46–49], lycopene [50], and polymers [51, 52] has further widened the industrial importance of *C*. *glutamicum*. Besides, *C*. *glutamicum* is also regarded as a model organism for the Corynebacterineae, such as the genus *Mycobacterium* [53].

**Fig 1:**
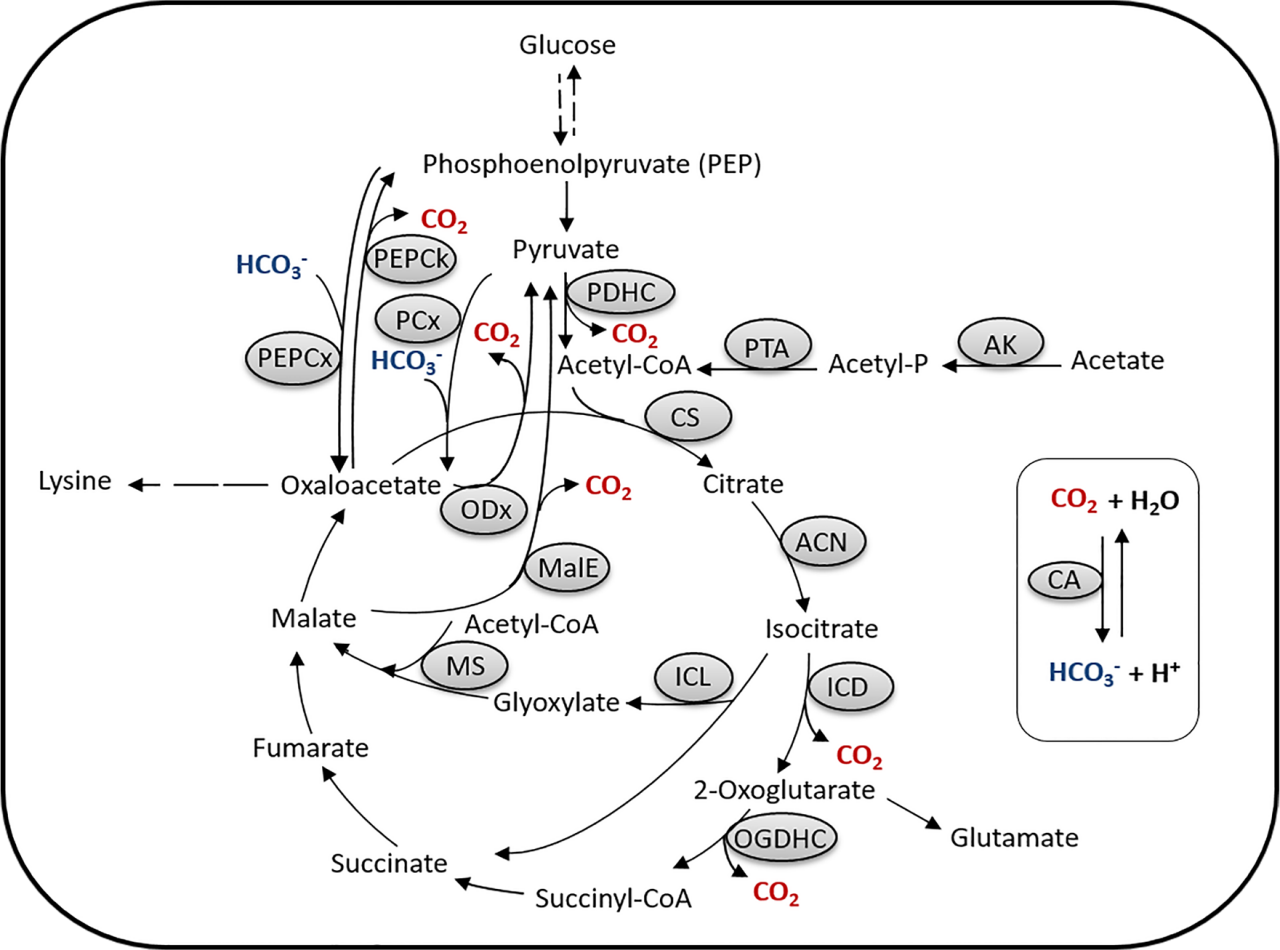
The phosphoenolpyruvate (PEP)-pyruvate-oxaloacetate node in *C*. *glutamicum*. Abbreviations: AK, acetate kinase; PTA, phosphotransacetylase; CA, carbonic anhydrase; CS, citrate synthase; ACN, aconitase; ICD, isocitrate dehydrogenase; OGDHC, oxoglutarate dehydrogenase complex; ICL, isocitrate lyase; MS, malate synthase; MalE, malic enzyme; ODx, oxaloacetate decarboxylase; PDHC, pyruvate dehydrogenase complex; PCx, pyruvate carboxylase; PEPCx, phosphoenolpyruvate carboxylase; PEPCk, phosphoenolpyruvate carboxykinase.

The PEP (phosphoenolpyruvate)-pyruvate-oxaloacetate node in *C*. *glutamicum* (Fig 1) has attracted specific attention due to its importance in carbon flux distribution within the central metabolism and in particular for supply of precursors required for the production of various amino acids (reviewed in [54, 4]), especially those of the aspartate and glutamate amino acid families. *C*. *glutamicum* possesses two C3-carboxylating anaplerotic enzymes, namely the PEP carboxylase and pyruvate carboxylase, converting phosphoenolpyruvate (PEP) and pyruvate to oxaloacetate, respectively [54]. Apart from these C3-carboxylating enzymes, *C*. *glutamicum* possesses three C4-decarboxylating enzymes, i.e., PEP carboxykinase, converting oxaloacetate to PEP, and oxaloacetate decarboxylase and malic enzyme, converting oxaloacetate and malate, respectively, to pyruvate (reviewed in [54]) (Fig 1). Whereas these decarboxylating enzymes [and also those of the tricarboxylic acid (TCA) cycle] liberate CO_2_, the carboxylating PEP and pyruvate carboxylases require HCO_3_^-^ as substrate [4, 17] which highlights the importance of intracellular CO_2_/HCO_3_^-^ balance for the central metabolism, especially the reactions at metabolic switch-points of carbon flux distribution. As HCO_3_^-^ is needed as substrate of metabolism, its significant source is the hydration of CO_2_. Due to the low tension of CO_2_ in the environment and its diffusion out of the cell, the spontaneously formed HCO_3_^-^ obviously is not sufficient to meet metabolic demands of the cell and thus, enzymatic hydration of CO_2_ might be necessary, especially under conditions when the intracellular CO_2_ generation is low [15, 17].

In *C*. *glutamicum*, two genes putatively coding for beta-type CA (β-CA) and gamma-type CA (γ-CA) have been identified and designated as *bca* (locus-tag cg2954) and *gca* (cg0155), respectively [17]. The *bca* gene is located between *mutY*, encoding an adenine glycosylase, and cg2953, encoding putatively a benzaldehyde dehydrogenase. The *gca* gene is directly preceeded by cg0154, encoding also a so far unknown protein and followed by *cysR*, encoding the dual transcriptional regulator CysR, which is involved in control of sulfur metabolism in *C*. *glutamicum* [55]. Though, a *gca-*deficient mutant of *C*. *glutamicum* did not show any phenotype under all conditions tested, a *bca-*deletion mutant showed no growth under normal atmospheric conditions (0.04% CO_2_) and this phenotype could be restored by increasing the CO_2_ concentration to 5% or by introducing a heterologous CA gene [17]. These results indicate that *bca* is functional as CA in *C*. *glutamicum*, that the *bca* gene product is essential and that the *gca* gene product is dispensable for growth of this organism under “normal” atmospheric conditions.

In this report, we analyzed the transcriptional organization of the *bca* and *gca* genes and investigated the transcriptional regulation of *bca* expression in glucose- or acetate-grown cells of *C*. *glutamicum*. We also constructed a *gca* deletion mutant of *C*. *glutamicum* and investigated the effect on growth and on expression of the *bca* gene.

## Materials and Methods

### Bacterial strains, plasmids, oligonucleotides and culture conditions

All bacterial strains and plasmids used in this study and their relevant characteristics and sources are given in **Table 1**, for oligonucleotides, their nucleotide sequence and purpose see **S1 Table** in the supplementary material.

**Table 1:** Strains and plasmids used in this study and their relevant characteristics.

Strain/plasmid	Relevant characteristic(s)	Source/ reference
**Strains**		
*E*. *coli* DH5α	*supE44*, *hsdR17*, *recA1*, *endA1*, *gyrA96*, *thi-1*, *relA1*	[56]
*E*. *coli* BL21 (DE3)	*ompT hsd*S_B_ (r_B_–m_B_–) *gal dcm* (DE3)	[57]
*E*. *coli* DH5α (pET2-P_*bca*_)	*E*. *coli* DH5α carrying plasmid pET2-P_*bca*_	This work
*E*. *coli* DH5α (pET2-P_*gca*_)	*E*. *coli* DH5α carrying plasmid pET2-P_*gca*_	This work
*E*. *coli* BL21 (DE3) (pET28-*ramA*)	*E*. *coli* BL21 (DE3) carrying plasmid pET28-*ramA*	[58]
*C*. *glutamicum* WT	Wild-type strain ATCC 13032	American Type Culture Collection
*C*. *glutamicum* (pET2-P_*bca*_)	*C*. *glutamicum* carrying plasmid pET2-P_*bca*_	This work
*C*. *glutamicum* DM1729 Δ*ilvB*	L-lysine-producer *C*. *glutamicum* DM1729 Δ*ilvB*	[59]
*C*. *glutamicum* DM1729 Δ*ilvB* (pET2-P_*bca*_)	*C*. *glutamicum* DM1729 Δ*ilvB* carrying plasmid pET2-P_*bca*_	This work
*C*. *glutamicum* (pET2-P_*gca*_)	*C*. *glutamicum* carrying plasmid pET2-P_*gca*_	This work
*C*. *glutamicum* Δ*atlR* (pET2-p4)	*C*. *glutamicum* Δ*atlR* carrying plasmid pET2-p4	C. Gabris, personal gift
*C*. *glutamicum* (pET2)	*C*. *glutamicum* carrying plasmid pET2	This work
*C*. *glutamicum* Δ*gca*	*C*. *glutamicum* with deleted *gca* gene in the genome	This work
*C*. *glutamicum* Δ*ramA*	*C*. *glutamicum* with deleted *ramA* gene in the genome	[58]
*C*. *glutamicum* Δ*ramA* (pET2-P_*bca*_)	*C*. *glutamicum* Δ*ramA* carrying plasmid pET2-P_*bca*_	This work
**Plasmids**		
pET2	Promoter probe vector, carrying the promoter-less *cat* reporter gene, Km^r^	[60]
pET2-P_*bca*_	pET2 carrying the *bca* promoter fragment cloned upstream of the *cat* reporter gene	This work
pET2-P_*gca*_	pET2 carrying the *gca* promoter fragment cloned upstream of the *cat* reporter gene	This work
pJET1.2/blunt	Linearized cloning vector, Amp^r^	CloneJET PCR Cloning kit (Thermo Scientific)
pJET1.2-*bca*-RACE	*bca* 5'-RACE product ligated into pJET1.2/blunt vector	This work
pJET1.2-*gca*-RACE	*gca* 5'-RACE product ligated into pJET1.2/blunt vector	This work
pET28-*ramA*	pET28 over-expression vector, carrying the *ramA* gene	[58]
pK19*mobsac*B vector	Km^r^, vector for integration of insert into the genome of the *C*. *glutamicum*	[61]
pK19*mobsac*B-Δ*gca*	pK19*mobsac*B vector carrying Δ*gca* insert	This work

*E*. *coli* was grown aerobically on 2×TY or TB complex medium [62] at 37°C as 5 ml-cultures in 15 ml-tubes or as 50 ml-cultures in 500 ml-baffled Erlenmeyer flasks on a rotary shaker at 120 rpm. Precultures of *C*. *glutamicum* were grown under the same conditions in 2×TY medium at 30°C. For preparation of solid plates, agar (18 g/l) was added to the medium. For the main cultures, cells of a *C*. *glutamicum* preculture were washed twice with 0.9% NaCl and added to freshly prepared minimal medium [63], containing 1% (w/v), 2% (w/v), 4% (w/v) glucose and/or 1% (w/v) acetate as carbon source(s). The cultures then were grown aerobically at 30°C as 50 ml-cultures in 500 ml baffled Erlenmeyer flasks on a rotary shaker at 120 rpm until the desired cell density was obtained. Plasmid-carrying strains were cultivated in the presence of kanamycin (50 μg/ml) or ampicillin (100 μg/ml). In fermentation experiments, the amino acid concentrations were determined by reversed-phase high-pressure liquid chromatography (RP-HPLC) as described before [26]. Growth of the *E*. *coli* and *C*. *glutamicum* cultures was followed by measuring the optical density at 600 nm (OD_600_).

### DNA preparation, manipulation and transformation

Restriction enzymes, T4 DNA ligase, Fast AP^TM^ thermosensitive alkaline phosphatase, DNase I, Maxima reverse transcriptase, terminal deoxynucleotidyl transferase and the CloneJet^TM^ PCR Cloning Kit were obtained from Thermo Scientific (Darmstadt, Germany), Phusion^®^ DNA polymerase from New England Biolabs (Ipswitch, MA, USA), *Taq* DNA polymerase from Genaxxon Biosciences (Ulm, Germany), and used as instructed by the manufacturer. The RNeasy Mini Kit and the HotStar polymerase kit was obtained from Qiagen (Hilden, Germany).

Plasmids from *E*. *coli* and *C*. *glutamicum* cells were isolated using the E.Z.N.A plasmid DNA Mini Kit (Omega Bio-tec Inc., Norcross, USA) or the method described in Green and Sambrook [62], respectively, and purified using the NucleoSpin Gel and PCR Clean-up Kit (Macherey-Nagel, Düren, Germany), according to the manufacturer’s instructions. Chromosomal DNA was isolated from *C*. *glutamicum* [64] and purified with phenol-chloroform purification method [62].

PCR experiments were performed in a Thermocycler (Biometra, Göttingen, Germany) using Phusion^®^ DNA or *Taq* DNA polymerase with oligonucleotides designed using the Clone Manager v.7 software and obtained from biomers.net (Ulm, Germany). All other reagents used for the PCR mix were obtained from Thermo Scientific. PCR products were purified using the NucleoSpin Gel and PCR Clean-up Kit from Macherey-Nagel.

Plasmid transfer into *C*. *glutamicum* was carried out by electroporation with an Electroporator 2510 (Eppendorf, Hamburg, Germany), and the recombinant strains were selected on 2xTY medium [62] agar plates containing kanamycin (50 or 15 μg/ml), as described by van der Rest et al. [65]. Electroporation of *E*. *coli* was carried out with competent cells according to the method of Dower et al. [66]. The success of the transformation was verified by plasmid preparation and/or other analyses indicated below.

### Cloning of the *bca* and *gca* promoter fragments

The promoter regions of the *bca* gene (position -500 to +20 with respect to the putative translational start site of *bca*) and of the *gca* gene (position -262 to +258 with respect to the putative translational start site of the upstream cg0154 gene) were amplified with primer pairs *bca*-promoter-fw/ -rev and *gca*-promoter-fw/ -rev, respectively. The two PCR products (i.e., the *bca* and *gca* promoter fragments) were separately ligated into the multiple cloning site of the promoter-probe vector pET2, upstream of the promoter-less *cat* reporter gene, encoding chloramphenicol acetyltransferase (CAT). The resulting plasmids pET2-P_*bca*_ and pET2-P_*gca*_ were transformed into *E*. *coli* DH5α cells, transformants were selected on 2xTY agar plates containing kanamycin. The success of the transformation was verified by plasmid preparation, restriction analysis, and sequence analysis (GATC Biotech, Konstanz, Germany) of the insert(s) in the isolated and purified plasmids, using vector-specific primers namely cm4 and pET-rev. Subsequently, the pET2-P_*bca*_ and pET2-P_*gca*_ plasmids were transformed into *C*. *glutamicum* by electroporation.

### RNA isolation and determination of the transcriptional start site

Total RNA was isolated from *C*. *glutamicum* carrying pET2-P_*bca*_ or pET2-P_*gca*_ plasmids, the transcriptional start sites (TSSs) were determined by cDNA synthesis and 5’ “**r**apid **a**mplification of **c**DNA-**e**nds” (5’-RACE) with PCR [67].

The *C*. *glutamicum* strains were grown in minimal medium with glucose 1% (w/v) as carbon source and harvested at the mid-exponential growth phase (OD_600_ of about 5) by centrifugation (4500 rpm for 10 minutes at 4°C). The total RNA was isolated as described by Auchter et al. [64] and after DNase I treatment purified using the RNeasy Mini Kit (Qiagen) according to the manufacturer’s instructions.

The cDNAs for the *bca* and *gca* genes were synthesized from purified total RNA by reverse transcription with pET2-specific primer cm5, using maxima reverse transcriptase according to the manufacturer’s instructions. Using terminal deoxynucleotidyl transferase, the cDNAs were subsequently tailed with poly-(A) at their 3ˊ**-**end with dATP. The poly-(A) tailed cDNAs then were amplified with primers oligo-(dT) and cm5, using the HotStar polymerase kit for PCR. The amplified PCR products were subsequently purified using the NucleoSpin Gel and PCR Clean-up Kit and ligated into the pJET1.2/blunt vector of the CloneJet^TM^ PCR Cloning Kit with blunt end ligation according to the manufacturer’s instructions, resulting in plasmids pJET1.2-*bca*-RACE and pJET1.2-*gca*-RACE. For both the *bca* and *gca* promoters, plasmids of three independent clones were sequenced using pJET1.2 vector-specific primers (pJET-fw and pJET-rev), sequence analysis was performed using the NCBI database and Clone Manager v.7 software.

### Enzyme Assays

For determination of specific CAT enzyme activities in cell extracts, *C*. *glutamicum* carrying pET2-P_*bca*_ or pET2-P_*gca*_ plasmid was grown in minimal medium containing glucose 1%, 2%(w/v) and/or acetate 1% (w/v) as carbon source, to the mid-exponential growth phase (OD_600_ of about 5) and cultures were harvested by centrifugation (4500 rpm, 4°C, 10 minutes). For preparation of cell extracts, the cell pellets were dissolved in 1 ml of washing buffer (200 mM Tris/ HCl pH 7.8), added to screw cap tubes containing 250 μl of glass-beads (diameter 0.1 mm) (Sigma Aldrich) and cell disruption was carried out in a Precellys 24 at speed 6.5 for 30 seconds three times with cooling on ice for 5 minutes each time. The glass-beads and cell debris were removed by centrifugation (14000 rpm for 30 minutes at 4°C). Protein quantification was performed using the Pierce BCA Protein Assay Kit (Thermo Scientific) in 96 well PS-Microplates, according to the manufacturer’s instructions. The specific CAT enzyme activities in the extracts were determined by the method described by Gerstmeir et al. [68].

### Over-production and purification of His_6_-RamA protein

The His_6_-RamA fusion protein was over-produced in *E*. *coli* BL21 (DE3) carrying pET28-*ramA* plasmid [58]. The culture was grown in 500 ml of TB medium in a 2 L Erlenmeyer flask and over-production of His_6_-RamA fusion protein was induced by addition of Isopropyl-β-D-thiogalactopyranoside (IPTG; 1 mM final concentration) after the culture reached an OD_600_ of 0.6 and was grown further for 4 hours to an OD_600_ of about 5. The over-produced His-tagged RamA fusion protein was purified on an ÄKTA^TM^ purifier (Amersham Biosciences, Freiburg, Germany) with a HisTrap^TM^ HP column (GE Healthcare, Uppsala, Sweden) using loading buffer (NNIG-20: 50 mM NaH_2_PO_4_, 300 mM NaCl, 20 mM imidazole, 5% glycerol (v/v), pH 8) and elution buffer (NNIG-500: 50 mM NaH_2_PO_4,_ 300 mM NaCl, 500 mM imidazole, 5% glycerol (v/v), pH 7.8).

For identification and verification of the purified His_6_-RamA, the protein sample was separated on a SDS-PAGE gel [69], the protein bands of interest were cut out of the gel (approximately 5 x 1.5 x 1 mm in size), and MALDI-TOF (Matrix Assisted Laser Desorption/ Ionization—Time Of Flight) analysis was performed as described by Gerstmeir et al. [68]. The MALDI-TOF analysis was done at the Forschungszentrum Jülich (Germany), the data obtained were analyzed using Mascot (PMF) Peptide mass fingerprint (http://www.matrixscience.com).

### Promoter binding assays with His-tagged RamA protein

The binding of purified His-tagged RamA protein with the *bca* promoter fragment P_*bca*_ and its sub-fragments PF1, PF2 and PF3 was tested using an electrophoretic mobility shift assay (EMSA). The fragment 1b (described in [58]) was used as negative control and an *aceA*-*aceB* intergenic fragment with known binding affinity for RamA [58] as positive control. Bovine serum albumin (BSA) was used as a negative protein control. The respective fragments were amplified by PCR with primers *bca*-promoter-fw and -rev, PF1-fw, PF2-fw, PF3-fw and *bca-*promoter-rev, 1b-fw and 1b-rev, and *aceA*-*aceB* intergenic-fw and *aceA-aceB* intergenic-rev, respectively. The products were purified using the NucleoSpin Gel and PCR Clean-up Kit. In the binding assays, about 70 ng of the fragments (each) were incubated for 20 minutes at room temperature with varying concentrations (0 to 2 μg) of His-tagged RamA protein in a total of 20 μl reaction mixture containing 10 mM Tris, 1 mM dithioerythritol, 1 mM EDTA, 1 μg Poly [d (I-C)] in 10% (v/v) glycerol. Afterwards, the mixture was separated on a 2% agarose gel in 1x TAE buffer (200 mM Tris-HCl, 100 mM acetate, 5 mM EDTA, pH 7.5) at 70 volts and stained with ethidium bromide.

### Construction of the *gca* deletion mutant in *C*. *glutamicum*

To construct a *gca* deletion mutant of *C*. *glutamicum*, the upper and lower regions (each 423 bp) of *gca* were generated by PCR using primer pairs Del-*gca*-upper-fw / -rev and Del-*gca*-lower-fw /–rev, respectively. The two products were purified using NucleoSpin Gel and PCR Clean-up Kit and subsequently combined in a cross-over PCR [70], using primer pair Del-*gca*-upper-fw / Del-gca-lower-rev, resulting in a truncated version of the *gca* gene with an intragenic deletion of 422 bp. The truncated *gca* gene then was ligated into the vector pK19*mobsac*B, resulting in plasmid pK19*mobsac*B-Δ*gca*. This plasmid was subsequently transformed into *C*. *glutamicum*. The replacement of the native *gca* (wild-type) gene with the truncated version in the genome of *C*. *glutamicum* was performed by homologous recombination (double crossover) according to the protocol described by Schäfer et al. [61]. The deletion/truncation of the chromosomal *gca* gene in the resulting *C*. *glutamicum* strain Δ*gca* was confirmed by colony PCR using primers *gca*-promoter-fw and Del-*gca*-lower-rev.

## Results

### Transcriptional start sites of the *bca* and *gca* genes in *C*. *glutamicum*

The transcriptional start sites (TSSs) of the *bca* and *gca* genes were determined using 5'-RACE analysis. For this purpose, the promoter regions of both genes were amplified, ligated into promoter-probe vector pET2 in front of the *cat* reporter gene, and the resulting promoter-reporter fusion plasmids pET2-P_*bca*_ and pET2-P_*gca*_, were transformed into *C*. *glutamicum*. The transformants were grown in minimal medium containing 1% (w/v) glucose and total RNA was isolated from cells harvested at the mid-exponential growth phase. cDNAs for the *bca-cat* and the *gca-cat* transcriptional fusions were synthesized, tailed with poly-(A), amplified and cloned into the pJET1.2/blunt cloning vector. For exact localization of the *bca* and *gca* TSSs, the amplified products were sequenced and analyzed.

**Fig 2:**
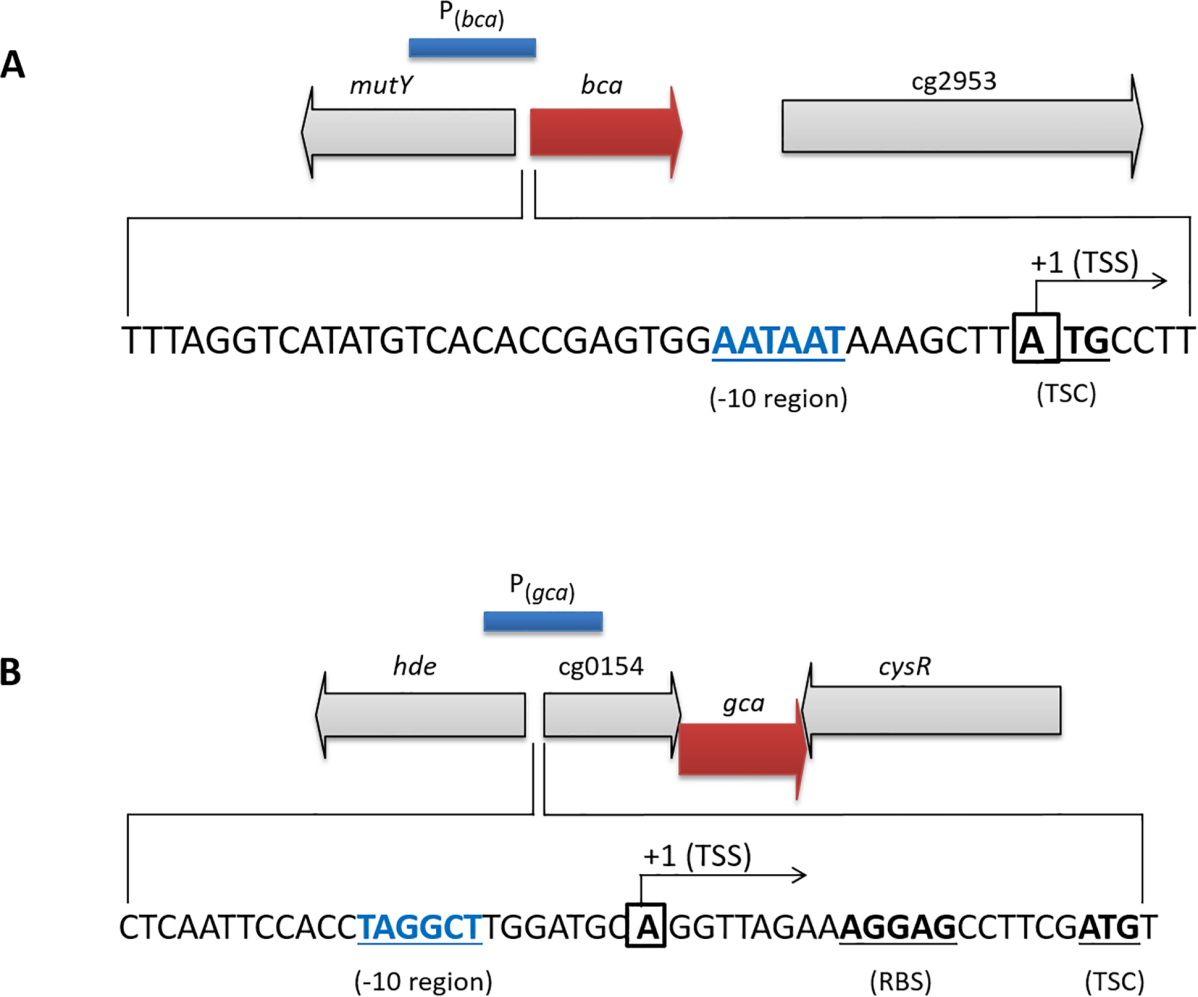
Transcriptional organization of the *bca* and *gca* genes in the genome of *C*. *glutamicum*. Genomic loci, promoter fragments used and transcriptional start sites (TSSs) of the *bca* **(A)** and *gca* **(B)** genes in *C*. *glutamicum*. The TSSs were identified by the 5'-RACE method. The putative -10 regions, the annotated translational start codon (TSC) of *bca* and of cg0154 and ribosome binding site (RBS) of cg0154 are indicated.

As indicated in Fig 2A, the *bca* TSS was found to be the first nucleotide “A” of the putative translational start codon (ATG), indicating that the *bca* gene codes for a leader-less transcript and thus, lacks a 5'-untranslated region. Centered 10 bp upstream of the TSS, an AATAAT motif was observed, which is very similar to the -10 consensus sequence for *C*. *glutamicum* [71].

The TSS for *gca* was identified to be an “A” residue at position—20 relative to the first nucleotide of the putative translational start codon (ATG) of cg0154, the gene located upstream of the *gca* (Fig 2B). In accordance, it has previously been shown that cg0154 and *gca* genes are co-transcribed [72]. Centered 10 bp upstream of the cg0154-*gca* TSS, the motif TAGGCT was observed, which shows reasonable similarity to the -10 consensus [TA(C/T)AAT] sequence for *C*. *glutamicum* [71]. Six bp upstream of the putative cg0154 translational start, we observed an AGGAG motif, which represents an ideal ribosomal binding site for *C*. *glutamicum* [72].

### Expression of the *bca* gene is subject to carbon source-dependent regulation

Using the *C*. *glutamicum* strains carrying plasmids pET2, pET2-P_*bca*_ and pET2-P_*gca*_, we performed a comparative expression analysis of the *bca* and *gca* genes in cells grown in minimal medium with 1% (w/v) glucose or 1% (w/v) acetate with initial pH values at 6.8 and 6.3. Cultures were harvested at the mid-exponential phase of growth (OD_600_ of about 5) and after cell disruption, the promoter activities were determined in the cell extracts by measuring the specific CAT activities. While the extracts from cells carrying the empty promoter-probe vector pET2 did not show any detectable CAT activity (< 0.01 U/mg protein), the extracts of the strains carrying the *bca* and *gca* promoters within pET2 showed activity and expression of *bca* also showed carbon source-dependent regulation (Table 2). The *bca* promoter activities were observed to be about 1.5- to 2-fold higher in extracts of glucose-grown cells as compared to that in extracts of acetate-grown cells. However, the *gca* promoter activities were very low, i.e., about 20-fold lower than that of the *bca* promoter in glucose- and in acetate-grown cells, indicating that expression of the *gca* gene in *C*. *glutamicum* is very low under the given conditions. Furthermore, it was observed that activities of the *bca* promoter were nearly the same on either glucose as carbon source on both pH values (6.3 and 6.8) or acetate as carbon source at both pH values (6.3 and 6.8) (Table 2). These results indicate that expression of the *bca* gene on either carbon source as well as carbon source-dependent regulation is independent of the initial pH 6.3 or 6.8.

**Table 2:** Specific chloramphenicol acetyltransferase (CAT) activities of different *C*. *glutamicum* strains carrying plasmids pET2-P_*bca*_ or pET2-P_*gca*_, cultured in minimal medium containing 1% or 2% (w/v) glucose and/or 1% (w/v) acetate with initial pH values of 6.3 or 6.8.

Strain	Minimal medium	Specific CAT activity [U/mg of protein]^a^
*C*. *glutamicum* (pET2-P_*bca*_)	+ glucose (pH 6.8)	1.60 ± 0.16
*C*. *glutamicum* (pET2-P_*bca*_)	+ acetate (pH 6.8)	0.71 ± 0.32
*C*. *glutamicum* (pET2-P_*bca*_)	+ glucose (pH 6.3)	1.60 ± 0.18
*C*. *glutamicum* (pET2-P_*bca*_)	+ acetate (pH 6.3)	1.08 ± 0.10
*C*. *glutamicum* (pET2-P_*gca*_)	+ glucose (pH 6.8)	0.09 ± 0.01
*C*. *glutamicum* (pET2-P_*gca*_)	+ acetate (pH 6.3)	0.05 ± 0.01
*C*. *glutamicum* DM1729 Δ*ilvB* (pET2-P_*bca*_)	+ glucose (pH 6.8)	1.43 ± 0.06
*C*. *glutamicum ∆ramA* (pET2-P_*bca*_)	+ glucose (pH 6.8)	2.38 ± 0.19
*C*. *glutamicum ∆ramA* (pET2-P_*bca*_)	+ glucose + acetate^b^	5.19 ± 0.06
*C*. *glutamicum* (pET2-P_*bca*_)	+ glucose + acetate^b^	1.37 ± 0.13
*C*. *glutamicum ∆gca* (pET2-P_*bca*_)	+ glucose (pH 6.8)	1.91 ± 0.06

^**a**^ The values are means of at least three independent experiments.

^**b**^ The initial pH values in these cultures were set to 6.3.

We also tested the *bca* promoter activity in cell extracts of the L-lysine-producing strain *C*. *glutamicum* DM1729 Δ*ilvB* transformed with plasmid pET2-P_*bca*_ and grown in minimal medium containing 2% glucose. As can be seen in Table 2, the specific CAT activity was in the same range as in extracts of *C*. *glutamicum* wild-type. In accordance, L-lysine production by *C*. *glutamicum* DM1729 Δ*ilvB* (pET2-P_*bca*_) was in the same range as previously reported [59] for the parental strain *C*. *glutamicum* DM1729 Δ*ilvB*, i.e., a final L-lysine concentration of about 32.3 mM after 24 h of incubation.

### Global regulator RamA negatively regulates expression of the *bca* gene

Based on the results of carbon source-dependent expression control of the *bca* gene in *C*. *glutamicum* with glucose and acetate (see above), we speculated the regulator of acetate metabolism RamA [58] to be involved in this regulation. RamA is a LuxR-type global regulator, essential for growth on acetate or ethanol and is involved in expression control of a variety of genes in central carbon metabolism [73]. The involvement of RamA in *bca* expression was tested by comparative *bca* promoter activity analysis with the wild-type and a RamA-deficient derivative of *C*. *glutamicum*. For this purpose, plasmid pET2-P_*bca*_ was transformed into *C*. *glutamicum* Δ*ramA* and the specific CAT activities of the resulting transformant and of *C*. *glutamicum* (pET2-P_*bca*_) were determined in cell extracts after growth of the cells in minimal medium with either glucose 1% (w/v) or glucose plus acetate (1% each, w/v) and harvested at the mid-exponential growth phase (OD_600_ of about 5). As shown in Table 2, the specific CAT activity and thus, the *bca* promoter activity in the Δ*ramA* mutant was about 1.6-fold higher in minimal medium with glucose and about four-fold higher with glucose plus acetate when compared to that in wild-type cells of *C*. *glutamicum*. The higher activity of the *bca* promoter in the absence of a functional RamA protein indicates that RamA acts as a negative transcriptional regulator for the expression of the *bca* gene in *C*. *glutamicum*.

**Fig 3:**
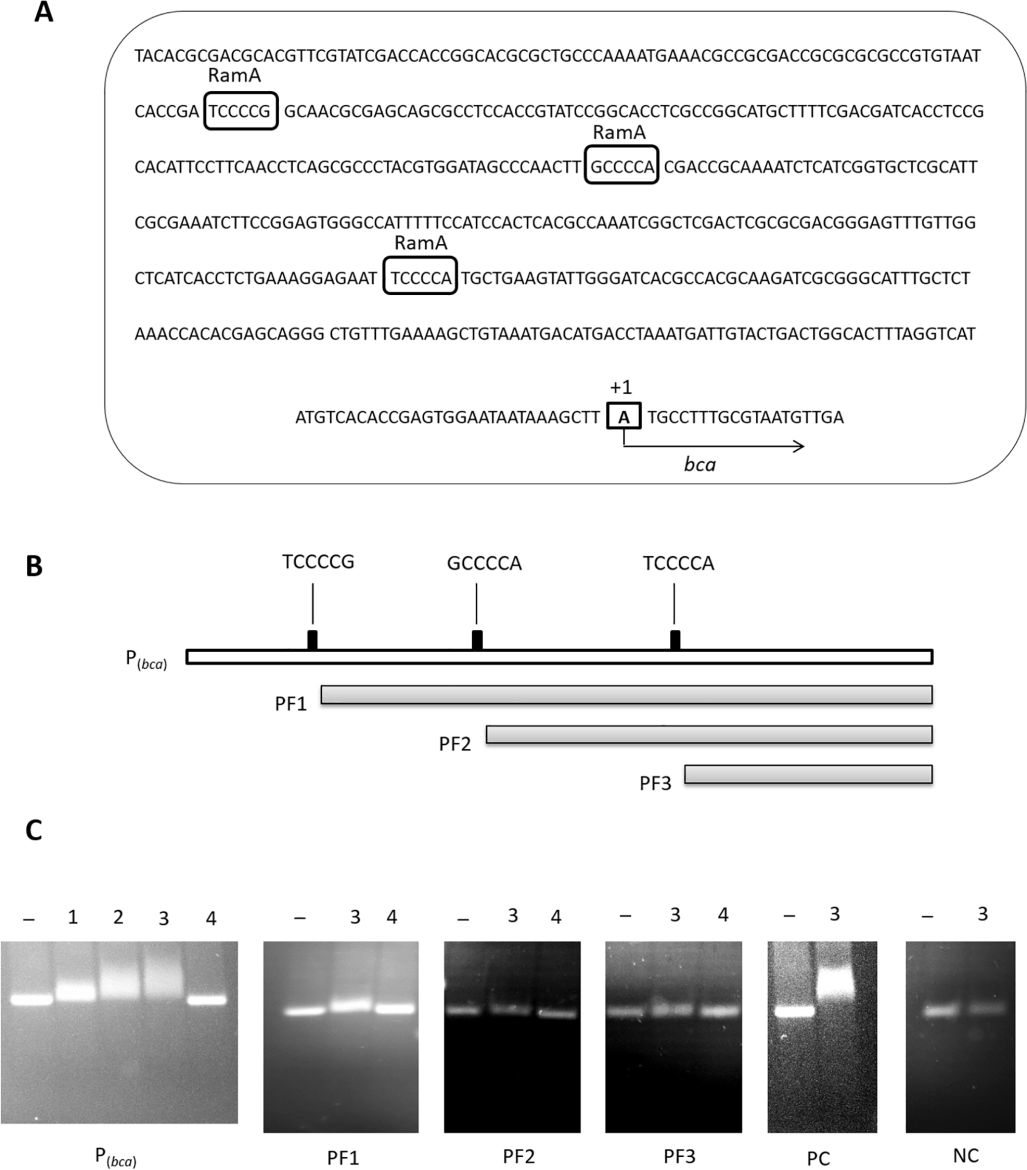
The *bca* promoter sequence and EMSAs. **(A):** Sequence of the *bca* promoter region (P_*bca*_) with its potential RamA binding motifs (boxed and indicated above the sequence) in *C*. *glutamicum*. The transcriptional start point is indicated by a box and “+1”. **(B):** The *bca* promoter (P_*bca*_) and its sub-fragments with exclusion of one, two and three RamA binding motifs in PF1, PF2 and PF3, respectively. **(C):** Representative EMSAs for binding assays using purified His- tagged RamA protein. The *aceA*-*aceB* inter-genic region was used as a positive control fragment (shown as PC) [58], fragment 1b [58] as a negative control fragment (shown as NC) and bovine serum albumin (BSA) as negative protein control. Lane–shows the respective fragment without protein whereas lanes 1, 2 and 3 show EMSAs using 0.8, 1.5 and 2 μg of His- tagged RamA, respectively, and lane 4 EMSAs using 2 μg of BSA instead of RamA.

The most common RamA binding motifs have been identified as A/T/C-GGGG-N and A/T/C-CCCC-N [73]. As shown in Fig 3A, three such motifs were also observed in the sequence of the *bca* promoter region and therefore, it was likely that the RamA protein binds to the *bca* promoter region. To analyze the binding of RamA to the *bca* promoter region, a His-tagged RamA protein was over-produced in *E*. *coli* BL21 (DE3) containing the pET28-*ramA* construct, identified by MALDI-TOF mass spectrometry, and used for EMSAs. For this purpose, the *bca* promoter fragment (P_*bca*_) and its sub-fragments with only two, one or no RamA binding motifs (PF1, PF2 and PF3, respectively, as shown in Fig 3B), were incubated with varying amounts (0–2 μg) of purified His-tagged RamA protein and the assay mixture was separated on an agarose gel. An *aceA*-*aceB* inter-genic fragment was used as positive control fragment for binding of the His-tagged RamA protein, as the RamA protein was already known to bind to this region [58], while fragment 1b having no binding affinity for RamA [58] was used as negative control fragment in the EMSA experiments. As shown in Fig 3C, the *bca* promoter fragment P_*bca*_ with all three RamA binding motifs was retarded by the RamA protein and the retardation was observed to be proportional to increasing concentration of the His-tagged RamA protein. The PF1 fragment showed less retardation with 2 μg RamA, fragments PF2 and PF3 did not show significant retardation. These results show that RamA binds to the *bca* promoter region and indicates that the two distal RamA binding motifs are functional.

Taken together, the higher *bca* promoter activity in *C*. *glutamicum* Δ*ramA* compared to that in the wild-type and binding of His-tagged RamA protein to the *bca* promoter fragment in the EMSA experiments showed that RamA negatively regulates the expression of the *bca* gene in *C*. *glutamicum*.

### Expression of the *bca* gene not affected in the absence of a functional Gca protein

To test for a possible effect of Gca on growth and on expression of the *bca* gene, a *gca* deletion mutant of *C*. *glutamicum* was generated, using the suicide vector pK19*mobsac*B and homologous recombination. A mutant version of *gca* gene was constructed by amplifying and condensing the *gca* upper and lower fragments (each 423 bp), resulting in a truncated *gca* gene with an intragenic deletion of 422 bp, which was exchanged with the native chromosomal copy of *gca*. In minimal medium containing glucose 4% (w/v), the resulting strain *C*. *glutamicum Δgca* was observed to grow with the same growth rates and to the same final OD_600_ as the wild-type *C*. *glutamicum* (data not shown), indicating that the *gca* gene is dispensable under the conditions tested. This result is consistent with the previous observation that *gca* is not essential for growth of *C*. *glutamicum* under normal conditions [17].

Plasmid pET2-P_*bca*_ was transformed into *C*. *glutamicum Δgca* and the activities of the *bca* promoter were determined by analysis of the specific CAT activities in crude cell extracts of cultures grown in minimal medium with glucose 1% (w/v). As shown in Table 2, the specific CAT activities in extracts of *C*. *glutamicum Δgca* were observed to be nearly the same as in the respective wild type derivative. This result indicates that there is no significant effect of the absence of a functional Gca protein on expression of the *bca* gene in *C*. *glutamicum*.

## Discussion

In nature, CO_2_ is in chemical equilibrium with HCO_3_^-^, carbonic acid and carbonate. Of these, CO_2_ and its hydrated counterpart HCO_3_^-^ are most important metabolites in living organisms as they serve as substrate or product in carboxylating and decarboxylation reactions, are involved in ion transport and internal pH regulation, regulate virulence and toxin formation in pathogenic bacteria and recently have been shown to be regulatory triggers of global transcriptional regulation and of microbial and mammalian production processes [74, 75, 4]. In aerobic (micro)organisms, CO_2_ is the product of respiration and as such in sufficient amounts present within metabolically active cells. However, for anaplerotic (and also other) carboxylation reactions, the physiologically most important reactant is HCO_3_^-^ [2, 15, 17]. As CO_2_ (but not HCO_3_^-^) can diffuse out of the cell (for a recent review see [76]), the intracellular conversion of CO_2_ to HCO_3_^-^ is essential to retain CO_2_ as genuine carboxylation substrate inside the cell. The chemical inter-conversion of CO_2_ and HCO_3_^-^ is relatively slow at physiological pH [77] and thus, nature has evolved enzymatic conversion by zinc-dependent CAs, catalyzing the reversible hydration of CO_2_ with high turnover numbers and allowing the cells to maintain the intracellular balance of CO_2_/HCO_3_^-^ that is needed for cellular processes [2, 78, 79]. The role of CAs has intensively been studied in *E*. *coli* and other microorganisms, in particular in relation to CO_2_/HCO_3_^-^ balance in the intracellular environment under physiological conditions (for references see introduction) and it has been observed that under atmospheric conditions, inactivation of CA(s) is lethal or highly inhibitory unless the CO_2_ content is increased to 5–10% [14–18, 80–82]. In spite of numerous studies on the physiological function of CAs in bacterial CO_2_/HCO_3_^-^ metabolism, there is much less information on the transcriptional organization and on expression control of the respective CA genes.

The purpose of this work was to broaden our knowledge about the transcriptional organization of the CA genes as well as expression analysis in relation to media composition (carbon sources) and transcriptional regulation in *C*. *glutamicum*. This organism is an industrial workhorse widely used for the production of amino acids and a variety of other metabolites [31, 32, 83, 84, 85, 86]. The PEP-pyruvate-oxaloacetate node of this organism (see Fig 1), being an important branch-point of carbon flux distribution and having a role in anaplerosis, gluconeogenesis and amino acid biosynthesis, involves several carboxylation/decarboxlation reactions and thus, a pivotal effect of the intracellular CO_2_/HCO_3_^-^ balance on the overall physiology of *C*. *glutamicum* can be presumed. Two CA genes, namely *bca* and *gca*, encoding β- and γ-CA, respectively, have previously been identified and *bca* has been shown to be essential under atmospheric conditions in *C*. *glutamicum* [17].

In this study, the TSS of the *bca* gene was identified to be the first nucleotide of its putative translational start codon (ATG). Thus, *bca* codes for a leaderless transcript which lacks the 5ˊ-untranslated region. Leaderless mRNAs starting with an AUG start codon have been reported in bacteria, archaea, eukaryotes [87] and also in *C*. *glutamicum* [71]. In fact, a recent RNA sequence analysis (RNAseq) with *C*. *glutamicum* revealed that about 33% of all mRNAs including that of the *bca* gene, in the cells are leaderless and that the translational start codon of these leaderless mRNAs generally is an AUG (about 79%) or GUG (about 21%) [72]. However, for leaderless mRNAs starting with the initiation codon AUG, no signals have been shown downstream of the 5ˊ-terminal AUG for recruitment of ribosomes [87]. In *E*. *coli*, it has been shown that for translation initiation of leaderless mRNAs, the molar ratio of the initiation factors IF2 and IF3 plays a final role, indicating that the translation efficiency of these mRNAs can be altered, based on the availability of components of the translational machinery [87, 88]. Homologues of genes encoding IF2 and IF3 have been found in the genome of *C*. *glutamicum* (cg1563 and cg2176, respectively; [89]), however, a role of these factors in translation of leaderless transcripts remains to be investigated.

Apart from the *bca* gene, we also determined the TSS for the *gca* gene, located at position -20 relative to the first nucleotide of the putative translational start codon of cg0154, a gene located upstream of the *gca*. Co-transcription of cg0154 and *gca* has previously been shown based on RNAseq analysis of *C*. *glutamicum* by Pfeifer-Sancar et al. [72].

To investigate a carbon source-dependent regulation of the CA genes in *C*. *glutamicum*, we investigated the expression of *bca* and *gca* in terms of their respective promoter activities in relation to glucose and acetate as carbon sources. This analysis revealed for both genes higher promoter activities when the cells were grown on glucose as compared to acetate. Furthermore, the activity of the *bca* promoter was observed to be about 15-fold higher than that of the *gca* promoter. The lower level of *gca* expression as compared to *bca* expression is consistent with the results of Mitsuhashi et al. [17] who found in Northern blot analysis of growing *C*. *glutamicum* cells the *gca* transcript level below the detection limit, suggesting that *gca* expression is either constantly very low or tightly regulated. Furthermore, it was also observed that expression of *gca* under control of the *lac* promoter restored the growth of *bca* mutant under normal environmental conditions [17]. However, our results suggest that the *bca* gene is subject to carbon source-dependent regulation as is the case for a variety of genes encoding key enzymes in central metabolism in *C*. *glutamicum* [53, 73, 90, 91].

The lower *bca* promoter activity and thus, the lower *bca* expression in acetate-grown cells than in glucose-grown cells of *C*. *glutamicum* might be attributed to a lower HCO_3_^-^ demand and reduced need of anaplerosis by pyruvate or PEP carboxylation and using the glyoxylate cycle for anaplerosis when growing on acetate instead of glucose. This hypothesis is in agreement with carbon flux analysis of the central metabolism of *C*. *glutamicum* growing in minimal medium containing glucose and/or acetate [92] and with the previous finding that a PEP and pyruvate carboxylase-deficient double mutant of *C*. *glutamicum* grows on acetate but not on glucose [93]. However the crucial role of *bca* during growth on glucose may not only be confined to replenishment of oxaloacetate or other TCA cycle intermediates as Mitsuhashi et al. [17] observed that addition of oxaloacetate, glutamate and succinate did not restore the growth of *C*. *glutamicum* Δ*bca*. It is, however, important to mention that *C*. *glutamicum* was found to be unable to take up and to grow on the TCA cycle intermediates fumarate, succinate and L-malate [94]. Therefore, a further potential role of CA in *C*. *glutamicum*, aside of replenishment of TCA cycle intermediates, has to be experimentally proven.

The transcriptional regulator RamA originally has been identified as the regulator of acetate metabolism in *C*. *glutamicum* [58]. Later it has been shown that RamA is functional as activator or as repressor in carbon metabolism of this organism and is involved in expression control of a variety of genes and operons encoding enzymes or pathways in the central metabolism of *C*. *glutamicum* (reviewed in [73, 90, 95]). Based on the observed carbon source-dependent regulation of the *bca* promoter, we speculated RamA to be involved in expression control of the *bca* gene. In fact, we found higher *bca* promoter activity in a RamA-deficient mutant of *C*. *glutamicum* when compared to the wild-type strain. This finding is in perfect agreement with our previous results of a genome-wide transcriptional profiling which showed an about 2.5-fold higher *bca* mRNA level in *C*. *glutamicum* Δ*ramA* (supplementary material in [73]). In addition, we found three typical RamA binding motifs in front of the *bca* TSS and showed binding of His-tagged RamA protein to the *bca* promoter region. All these results indicate RamA to be a negative transcriptional regulator for expression of *bca* in *C*. *glutamicum*. However, based on the differential expression of *bca* in glucose-grown and in glucose-plus-acetate-grown cells of the RamA-deficient mutant, it can be suggested that other transcriptional regulator(s) and/or effector(s) are involved directly or indirectly in the carbon source-dependent *bca* regulation. This argument is also reinforced by the fact that RamA affects and/or is affected by other transcriptional regulators such as GlxR, SugR and RamB [58, 68, 91].

## Supporting Information

S1 Table Oligonucleotides used in this study. (DOCX)
