# Supplementary material for: Transcriptional Regulation of the β-Type Carbonic Anhydrase Gene bca by RamA in Corynebacterium glutamicum
Source: PLoS One. 2016 Apr 27;11(4):e0154382. doi: 10.1371/journal.pone.0154382 (PMC4847777; doi:10.1371/journal.pone.0154382)
Supplement: S1 Table — (DOCX) [file pone.0154382.s001.docx]

**Supplementary material**

**S1 Table. Oligonucleotides used in this study.**

| **Oligonucleotides** | **Sequence** | **Purpose** |
| --- | --- | --- |
| *bca*-fw | 5**'**-GGGAATTCCATATGCCTTTGCGTAATGTTG-3**'** | Fw primer for amplification of *bca* gene |
| *bca*-rev | 5**'**-ACGCGTCGACCTAACCCACGTTCTTGCTAA-3**'** | Rev primer for amplification of *bca* gene |
| *gca*-fw | 5**'**-GGGAATTCCATATGACACCTCAACCACTGAT-3**'** | Fw primer for amplification of *gca* gene |
| *gca*-rev | 5**'**-ACGCGTCGACTTACTCTCGTACCCTAACCTC-3**'** | Rev primer for amplification of *gca* gene |
| *bca*-promoter-fw | 5**'**-ACGCGTCGACTACACGCGACGCACGTTCGT-3**'** | Fw primer for *bca* promoter (SalI) |
| *bca*-promoter-rev | 5**'**-CGGGGTACCTCAACATTACGCAAAGGCATAAGC-3**'** | Rev primer for *bca* promoter (KpnI) |
| *gca*-promoter-fw | 5**'**-ACGCGTCGACCTCACCGGCACCCCAGCAAT-3**'** | Fw primer for *gca* promoter (SalI) |
| *gca*-promoter-rev | 5**'** -CGGGGTACCGCCCCTGTAGTCAGCCGCGA-3**'** | Rev primer for *gca* promoter (KpnI) |
| cm4 | 5**'** -GAAAATCTCGTCGAAGCTCG-3**'** | pET2 vector-specific primer |
| cm 5 | 5**'** -AAGCTCGGCGGATTTGTC-3**'** | pET2 vector-specific primer |
| pET2-rev | 5**'** -CACACCATAGTGGCCATGAG-3**'** | pET2 vector-specific primer |
| Oligo-(dT) | 5**'** GACCACGCGTATCGATGTCGACTTTTTTTTTTTTTTTT-3**'** | Primer for poly-(A) tail of cDNA |
| pJET-fw | 5**'** -CGACTCACTATAGGGAGAGCGGC-3**'** | pJET vector-specific fw primer |
| pJET-rev | 5**'** -AAGAACATCGATTTTCCATGGCAG-3**'** | pJET vector-specific rev primer |
| *aceA*-*aceB* intergenic-fw | 5**'** -AGTCTGAGCAGACAACAGTTCCTG-3**'** | Fw primer for *aceA*-*aceB* inter-genic region |
| *aceA*-*aceB* intergenic-rev | 5**'** -ACGTGGCTTTCCAACGTTTGACAT-3**'** | Rev primer for *aceA*-*aceB* inter-genic region |
| 1b-fw | 5**'** -TGTGCTGCTCAAAGCGTG-3**'** | Fw primer for 1b fragment |
| 1b-rev | 5**'** -ATGACACTTCAGGCTTGTGCCT-3**'** | Rev primer for 1b fragment |
| PF1-fw | 5’-ACGCGAGCAGCGCCTCCACC-3’ | Fw primer for PF1 fragment of *bca* promoter |
| PF2-fw | 5**'** -CCGCAAAATCTCATCGGTGC-3**'** | Fw primer for PF2 fragment of *bca* promoter |
| PF3-fw | 5**'** -TGAAGTATTGGGATCACGCC-3**'** | Fw primer for PF3 fragment of *bca* promoter |
| Del-*gca*-upper-fw | 5**'** -TGCACTGCAGTCAGGACATGGTTGATTATGT-3**'** | Fw primer for upper fragment of *gca* deletion (PstI) |
| Del-*gca*-upper-rev | 5**'** -GTACCGTAATCGGCAATCCATGCGCTTTCATGGAT-3**'** | Rev primer for upper fragment of *gca* deletion |
| Del-*gca*-lower-fw | 5**'** -CGATTACGGTACAGAAACATCAAAAGCCCAGGCTT-3**'** | Fw primer for lower fragment of *gca* deletion |
| Del-*gca*-lower-rev | 5**'** -TGCACTGCAGAGCAGGGTTTCTGATCAGGGTATC-3**'** | Rev primer for lower fragment of *gca* deletion (PstI) |
| pK19*mobsac*B-fw | 5**'** -TAATGCAGCTGGCACGAC-3**'** | pK19*mobsac*B vector- specific fw primer |
| pK19*mobsac*B-rev | 5**'** -GTAGCTGACATTCATCCG-3**'** | pK19*mobsac*B vector- specific rev primer |
